# Yoga and High-Intensity Interval Training Show Comparable Effects on HbA1c in Type 2 Diabetes: A Systematic Review and Preliminary Pilot Network Meta-Analysis in Adult Populations

**DOI:** 10.3390/healthcare14121703

**Published:** 2026-06-15

**Authors:** Saw Ye Win Thu, Sneha Patnaik, Yin-Hwa Shih

**Affiliations:** Department of Healthcare Administration, Asia University, Taichung 41354, Taiwan; sawyewinthu09@gmail.com (S.Y.W.T.); sneha.patnaik@gmail.com (S.P.)

**Keywords:** T2DM, high-intensity interval training, resistance training, yoga, HbA1c

## Abstract

**Background/Objectives**: Exercise is pivotal for glycemic control in type 2 diabetes mellitus (T2DM), yet the relative efficacy of various exercise modalities remains inconclusive. This network meta-analysis aimed to evaluate and provide a preliminary ranking of exercise interventions on HbA1c levels in adults with type 2 diabetes mellitus, to facilitate clinically relevant network comparisons and to generate evidence for future large-scale comparative trials. **Methods**: A systematic review and network meta-analysis were conducted in accordance with PRISMA guidelines. Electronic databases (PubMed, MEDLINE, Cochrane Library, CINAHL, and ProQuest) were searched from inception to Dec 2024. Randomized controlled trials evaluating exercise interventions in adults with T2DM were included. Risk of bias was assessed independently by two reviewers using the JBI critical appraisal tool. The primary outcome was the change in HbA1c level. **Results**: Six randomized controlled trials involving a total of 511 participants (256 in the treatment group and 255 in the control group) were included in the final analysis. Both high-intensity interval training (MD = −0.322; 95% CI: −0.559 to −0.084; *p* = 0.008) and yoga (MD = −0.366; 95% CI: −0.534 to −0.198; *p* < 0.001) significantly reduced HbA1c compared with the active control. Although the preliminary ranking analysis suggested a higher probability of effectiveness for yoga (SUCRA 1) than for HIIT (SUCRA 0.5), the indirect comparison revealed no statistically significant difference in HbA1c reduction between the two interventions (MD = −0.044; 95% CI: −0.335 to 0.247; *p* = 0.766). **Conclusions**: These findings provide preliminary, evidence-generating; however, given the sparse network and absence of head-to-head trials, the treatment hierarchy should be interpreted with extreme caution and selected based on patients’ preferences and tolerance. Registration: PROSPERO [CRD42025650162].

## 1. Background

Type 2 diabetes mellitus (T2DM) is a chronic metabolic disorder characterized by persistent hyperglycemia due to insulin resistance and impaired insulin secretion [1]. It continues to pose a substantial global health burden and is associated with an increased risk of cardiovascular disease. In 2024, an estimated 588.7 million adults aged 20–79 years were living with diabetes (11.1% of the global population), accounting for 579.5 million cases (98.4%). It is projected to rise to 852.5 million cases by 2050, reflecting a 17% increase driven by population aging and increasing urbanization worldwide [2]. Rapid urbanization, sedentary behavior, and dietary transitions have accelerated the growing burden of disease [3]. Alongside pharmacological interventions, lifestyle modification is essential for effective T2DM management [4].

Physical activity is one of the most effective non-pharmacological strategies for prediabetes and T2DM, enhancing insulin sensitivity and thereby improving glycemic regulation [5]. Evidence indicated that aerobic exercise (AE) [6,7], resistance training (RT), and combined training (CT) all improved glycemic control, with CT demonstrating the greatest reduction in fasting blood glucose (FBG) [8,9]. However, exercise dosage was critically associated with a reduction in HbA1c [10].

In addition to traditional exercise modalities, mind–body practices such as yoga and tai chi have gained increasing recognition for their beneficial effects on glycemic regulation, particularly for individuals who find high-intensity exercise challenging, undesirable, or excessively demanding. Yoga, originating in India, has been shown to improve the regulation of blood glucose [11]. Tai chi, a traditional Chinese mind–body exercise emphasizing slow, mindful movements and controlled breathing, demonstrated beneficial glycemic control, particularly beneficial for older adults with T2DM who were at increased risk of falls [12]. Moreover, tai chi has been reported to be superior to brisk walking in reducing blood glucose and glycated hemoglobin across studies [13].

Given the lack of head-to-head trials and the heterogeneity in exercise modalities, intensities, and outcomes reported across existing studies, the comparative effectiveness of different exercise interventions for glycemic control in individuals with T2DM remains unclear. To support clinically relevant network comparisons, we conducted a preliminary pilot network meta-analysis of a range of exercise interventions, including high-intensity interval training (HIIT) and mind–body practices. Our objective was to evaluate and provide a preliminary ranking of exercise interventions on HbA1c levels in adults with type 2 diabetes mellitus, to facilitate clinically relevant network comparisons and to generate evidence for future large-scale comparative trials.

## 2. Methodology

### 2.1. Methodological Standards and Registration

The study was performed in accordance with the Preferred Reporting Items for Systematic Reviews and Meta-Analyses (PRISMA) guidelines. The protocol was registered in the International Prospective Register of Systematic Reviews (PROSPERO; registration number: CRD42025650162).

### 2.2. Search Strategy

A systematic literature search was conducted across five electronic databases: CINAHL, the Cochrane Library, MEDLINE, PubMed, and ProQuest. The search strategy was constructed based on the PICO framework, using controlled vocabulary and free-text terms for type 2 diabetes mellitus (T2DM), hyperglycemia, physical exercise, aerobic exercise, resistance training, walking, running, high-intensity interval training (HIIT), yoga, tai chi, HbA1c, glycemic control, postprandial glucose (PPG), fasting blood glucose (FBG), insulin sensitivity, body mass index (BMI), body weight, blood pressure, waist circumference (WC), and blood glucose. Detailed search terms and strategy were provided in Supplementary Data S1.

### 2.3. Study Selection

Following database searches, all retrieved articles were exported from each database into EndNote X9, and duplicates were removed. Titles and abstracts were independently screened by two reviewers, after which full-text articles were assessed for eligibility in accordance with the PRISMA guidelines. Studies were excluded if they met any of the following criteria: (1) lacked a control group; (2) did not specifically include individuals with type 2 diabetes mellitus (T2DM); (3) were published in languages other than English; or (4) were review articles, editorials, expert opinions, or meta-analyses. Any discrepancies between reviewers (SYWT and SN) were resolved through discussion or adjudication by a third reviewer (YHS).

### 2.4. Quality Assessment and Data Extraction

Two authors independently assessed the methodological quality and risk of bias of the included studies using the Joanna Briggs Institute (JBI) Critical Appraisal Tool for randomized controlled trials (RCTs) [14]. This tool evaluates specific methodological features of clinical trials, including randomization procedures, allocation concealment, and the blinding of participants, personnel, and outcome assessors. The quality thresholds applied (<49% high risk, 50–69% moderate risk, and >70% low risk) were adopted from established literature [15] to provide a standardized and objective categorization of the risk of bias across the included RCTs, ensuring consistency with established methodological standards. Overall, 2 studies (6.9%) were rated as having a high risk of bias, 10 studies (34.5%) as moderate risk, and the remaining 17 studies (58.6%) as low risk of bias, suggesting that 93.1% have moderate or low risk of bias (Supplementary Table S1). Although the relatively small proportion of high-risk studies may suggest a generally acceptable level of methodological quality, the potential influence of study limitations should be cautious when interpreting the pooled estimates and treatment rankings, particularly given the analysis’s preliminary nature.

Data extraction was performed independently using a standardized form and included (1) study characteristics: author, year of publication, country, study design, population characteristics, mean age, sample size, intervention groups, type of exercise intervention, and intensity monitoring; (2) outcomes, mean values, and standard deviations of HbA1c. All available full-text and Supplementary Materials were carefully examined for the mean and standard deviation of HbA1c change. Studies with missing or non-extractable data, inconsistent outcome reporting, or insufficient network connectivity were included in the qualitative (narrative) synthesis summarizing all 29 studies but were excluded from the quantitative network meta-analysis.

### 2.5. Network Meta-Analysis

Initial pairwise meta-analyses examining the effects of exercise interventions on HbA1c were conducted using Comprehensive Meta-Analysis (CMA) software version 2 (Biostat, Englewood, NJ, USA). Heterogeneity was assessed using the Q test and I^2^ statistic. Interventions demonstrating significant reductions in HbA1c were subsequently included in a network meta-analysis (NMA) performed using Stata 18 (StataCorp, College Station, TX, USA).

Because no direct head-to-head trials comparing high-intensity interval training (HIIT) and yoga were identified, NMA was used to facilitate indirect comparisons through a common comparator (active control). Arm-level data (study, treatment, mean change, standard deviation of change, and sample size) were entered using the network setup command (Supplementary Table S4). Analyses were based on change-from-baseline HbA1c values derived from reported pre- and post-intervention summary statistics. No imputation methods or assumed correlation coefficients were applied.

Treatment effects were synthesized using a random-effects model with the restricted maximum-likelihood (REML) estimator to account for between-study heterogeneity. Pooled mean differences (MDs) and corresponding 95% confidence intervals (CIs) were estimated relative to the common reference group.

The transitivity assumption was assessed by examining potential effect modifiers across studies, including baseline HbA1c levels, intervention duration, exercise intensity, and control group conditions. Although some variation existed, these characteristics were considered reasonably comparable across treatment comparisons, supporting the plausibility of indirect comparisons. Nevertheless, residual clinical heterogeneity cannot be excluded and should be considered when interpreting the findings.

The treatment network was sparse, contained no closed loops, and included no multi-arm trials. Therefore, indirect estimates were derived from independent studies connected through the common comparator, and covariance between treatment effect estimates was assumed to be zero. Formal inconsistency testing was not possible; consequently, the results should be interpreted as exploratory and hypothesis-generating.

Potential publication bias and small-study effects were explored using a comparison-adjusted funnel plot (Supplementary Table S3). Given the sparse network structure, the funnel plot was interpreted descriptively.

Network meta-analysis results were presented using network plots, forest plots, league tables, and Surface Under the Cumulative Ranking Curve (SUCRA) values. SUCRA values range from 0% to 100%, with higher values indicating a greater probability of ranking among the most effective interventions. Because treatment rankings in sparse networks may be unstable, SUCRA results were interpreted cautiously and considered exploratory.

## 3. Results

### 3.1. Screening Result

**Figure 1:**
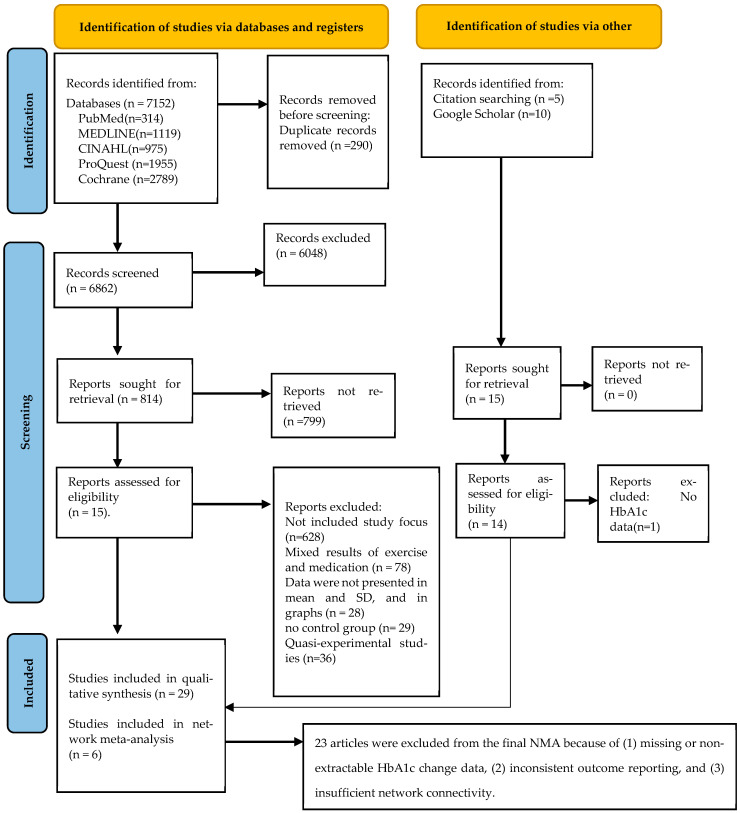
PRISMA 2020 flow diagram of study identification, screening, eligibility, and inclusion.

A total of 7152 records were identified through searches of five electronic databases, and an additional 15 records were identified through manual searching. After removing 290 duplicate records, 6862 articles remained for title and abstract screening. Of these, 6048 articles were excluded, leaving 814 articles for full-text review. Following full-text assessment, 799 articles were excluded because they did not meet the study objectives, reported mixed effects of exercise and medication, or failed to provide outcome data in the form of means and standard deviations. Ultimately, 29 studies met the inclusion criteria for the systematic review. Of these, 23 studies were excluded from the network meta-analysis due to missing or non-extractable HbA1c data, inconsistent reporting of outcomes, or insufficient connections within the treatment network (Figure 1).

### 3.2. Characteristics of the Included Study

All included studies were randomized controlled trials (RCTs). The sample size of the intervention groups ranged from 10 participants [16,17] to 163 participants [18], while the control size ranged from 9 [16] to 219 participants [19]. A total of 29 studies were included, comprising two studies from Egypt [20,21], one from the United Arab Emirates [19], one from Saudi Arabia [22], six from India [23,24,25,26,27,28], one from the United Kingdom [29], eight from China [13,18,30,31,32,33,34,35], one from Turkey [36,37,38], two from Japan [37,38], one from the United States [39], two from Iran [16,17], one from Portugal [40], one from Sri Lanka [41], one from Australia [42] and one from Indonesia [43].

Eleven studies investigated high-intensity interval training or moderate-intensity continuous training (HIIT/MICT) [16,20,21,22,29,31,36,39,40,42,43]; four examined tai chi [13,30,32,33]; six evaluated yoga interventions [24,25,26,27,28,37]; and eight focused on structured exercise programs combined with resistance training [17,18,19,23,34,35,38,41]. The intervention duration across studies ranged from 10 to 48 weeks. Outcomes were assessed at baseline and at the end of the intervention period, with measurement protocols varying among studies.

Exercise intensity was monitored using ergometers, Swedish Monark cycle ergometers, electronic treadmills, and electromagnetic bicycles. Resistance training protocols employed small equipment, such as dumbbells, resistance bands, and kettlebells, for exercises including seated rows, chest presses, leg presses, and planks, as well as weight-training machines for bench press, lat pulldown, biceps curl, and rowing. Aerobic exercise interventions included stretching, walking, stepping, and cycling. Yoga interventions primarily emphasized breathing techniques, meditation, and relaxation. Tai chi interventions included Chen-style tai chi, focusing on breathing, flexibility, stress reduction, and balance, as well as the 24-form Yang-style tai chi, which incorporated coordinated physical movements and breathing exercises. Articles included in the network meta-analysis were presented in Table 1, whereas studies included in the qualitative analysis were summarized in Table S2.

### 3.3. Evaluation of the HbA1c Reduction Effect Using Preliminary Meta-Analysis

Firstly, we categorized studies based on the PICO criteria, the primary outcome, and the similarity of control groups. Intervention classifications were based on the descriptions provided in the original articles and grouped according to their primary exercise characteristics.

**Figure 2:**
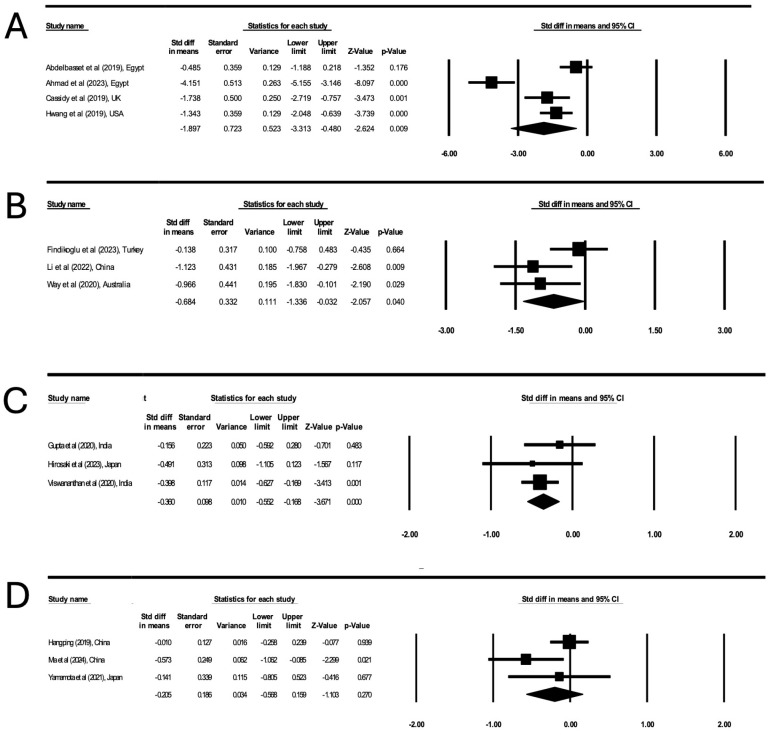
Forest plot of the effects of different exercise modalities on HbA1c improvement (preliminary meta-analysis). (**A**) High-intensity interval training compared with usual care [20,21,29,39]. (**B**) High-intensity interval training compared to active control [31,36,42]. (**C**) Yoga compared to active control [25,28,37]. (**D**) Resistance training compared to usual care [18,34,38].

Based on the consistency of outcomes, availability of comparable data, and methodological quality, 13 studies with comparable characteristics and at least three eligible trials were selected, grouped, and initially analyzed using meta-analysis. (Figure 2). The HIIT compared to usual care (SMD = −1.897, 95%: −3.313 to −0.480, *p* = 0.009) or active control (SMD = −0.684, 95%: −1.336 to −0.032, *p* = 0.009), and yoga compared to active control (SMD = −0.38, 95%: −0.562 to −0.166, *p* < 0.001) showed a significant effect on HbA1c reduction. The RT pool effect showed no significant difference compared with usual care (SMD = −0.205, 95% CI: −0.568 to 0.159, *p* = 0.27) (Figure 2).

### 3.4. Comparison of the HbA1c Reduction Between HIIT and Yoga in Network Meta-Analysis

**Figure 3:**
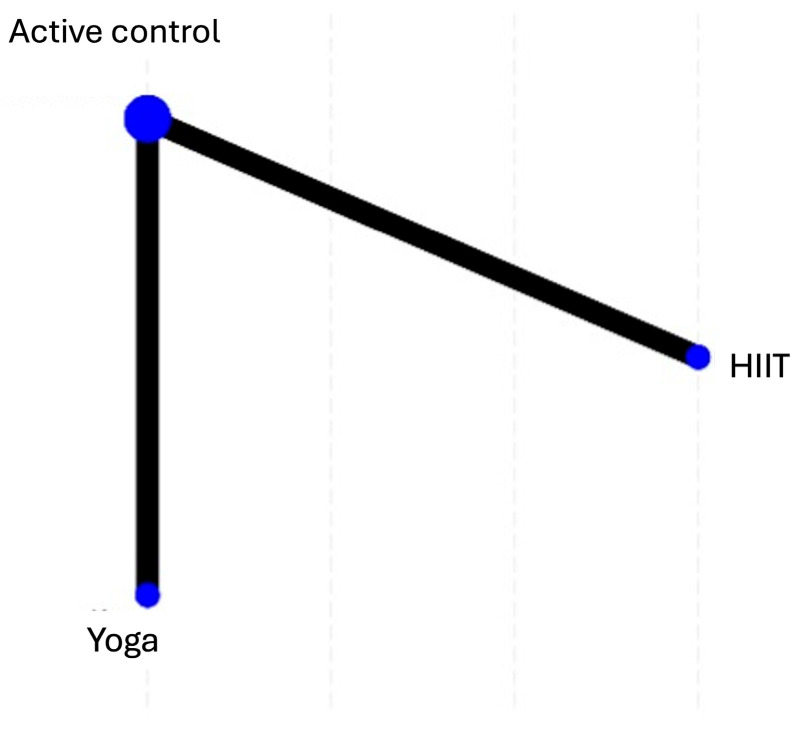
Network plot of exercise interventions included in the network meta-analysis. Each node represents an intervention (HIIT, yoga, and active control) [25,28,31,36,37,42], with the node size proportional to the number of participants receiving that treatment. The connecting lines indicate direct comparisons between interventions, and their thickness corresponds to the number of studies comparing the respective treatments.

Subsequently, studies evaluating HIIT and yoga compared with active control were included in the network meta-analysis [25,28,31,36,37,42]. A total of six randomized controlled trials were included, comprising three interventions: HIIT, yoga, and an active control. The network plot demonstrated a connected three-node structure with active control serving as the reference intervention. Most studies provided direct evidence for comparisons between active control and HIIT, or between active control and yoga, whereas the contrast between HIIT and Yoga was primarily based on indirect evidence (Figure 3).

### 3.5. Relative Treatment Effects on HbA1c Reduction

**Figure 4:**
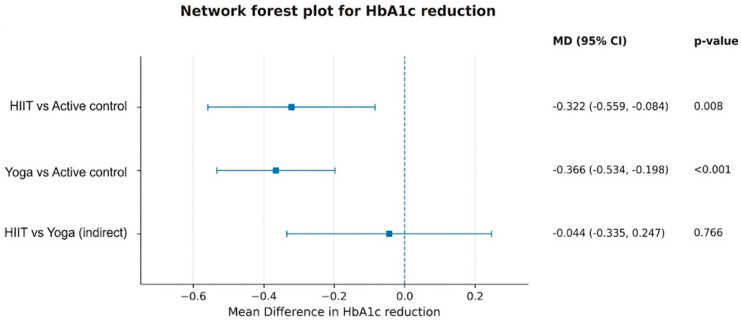
Prediction interval of the effects of exercise modalities on HbA1c reduction.

A multivariate random-effects model was fitted to evaluate network consistency (Table 2). The between-study variance was negligible (τ = 2.91 × 10^−12^), suggesting minimal heterogeneity across studies and supporting the assumption of model consistency. HIIT (MD = −0.322, 95% CI: −0.559, −0.084, *p* = 0.008) and yoga (MD = −0.366, 95% CI: −0.534, −0.198, *p* < 0.001) were significantly more effective than active control in improving HbA1c level. The indirect comparison between HIIT and yoga showed no statistically significant difference (MD = −0.044, 95% CI: −0.335 to 0.247, *p* = 0.766) (Table 2 and Figure 4). Visual inspection of the comparison-adjusted funnel plot did not reveal substantial asymmetry (Supplementary Table S3). Because the network included only a limited number of studies and lacked closed loops, the interpretation of publication bias and the formal assessment of inconsistency were limited.

### 3.6. Relative Treatment Effects and Ranking Probabilities

Compared with active control, both HIIT and yoga significantly reduced HbA1c levels. The mean difference (MD) for HIIT versus active control was 0.322 (95% CI: 0.084 to 0.559), while the MD for yoga versus active control was 0.366 (95% CI: 0.198 to 0.534). An indirect comparison between HIIT and yoga revealed no significant difference in HbA1c reduction, with an MD of −0.044 (95% CI: −0.335 to 0.247). Although yoga showed a numerically greater reduction in HbA1c than HIIT, the difference between the two interventions was not statistically significant (Table 3).

Ranking probability analysis suggested that yoga had a higher probability of being the most effective intervention for HbA1c reduction, with a probability of 1.00 for being ranked first and a SUCRA value of 1.00. HIIT was consistently ranked second, with a probability of 1.00 for being the second-best intervention and a SUCRA value of 0.50. Active control was ranked as the least effective intervention, with a probability of 1.00 for being ranked third and a SUCRA value of 0.00 (Table 4). However, it is essential to note that SUCRA rankings are probabilistic estimates and do not imply definitive clinical superiority, particularly as the indirect comparison between yoga and HIIT showed no statistically significant difference (*p* = 0.766).

## 4. Discussion

This study addressed an important gap in the literature where direct head-to-head comparisons between HIIT and yoga are lacking. Using a network meta-analysis framework, both HIIT and yoga were associated with significant reductions in HbA1c compared with an active control. Although yoga had the highest probability and SUCRA values, the indirect comparison between yoga and HIIT was not statistically significant. Therefore, the ranking results should be interpreted as probabilistic and exploratory rather than definitive evidence of superiority. Because all included studies involved adults with type 2 diabetes mellitus and were connected through a common comparator, the transitivity assumption was considered reasonably plausible. Nevertheless, residual heterogeneity may have influenced the indirect comparison, and the findings should be interpreted with caution.

The beneficial effects of HIIT on glycemic control are believed to result primarily from improved insulin sensitivity and enhanced skeletal muscle glucose uptake [21,29,44,45]. In the included studies, exercise intensity was prescribed using objective physiological measures, such as heart rate or VO_2_max, which may have improved the consistency of intervention delivery. In addition, the shorter time commitment of HIIT may make it more practical for some individuals. Yoga may improve glycemic control through multiple mechanisms [46,47,48], including enhanced insulin sensitivity, increased glucose uptake, and attenuation of hypothalamic–pituitary–adrenal axis activation, thereby reducing cortisol levels and improving metabolic regulation [49,50]. Previous studies have reported glycemic benefits of yoga comparable to those achieved with conventional exercise programs [51,52], suggesting that yoga may represent a feasible alternative exercise modality for selected patients.

Considerable heterogeneity existed across studies, including differences in exercise intensity, intervention duration, participant characteristics, and intervention protocols. Yoga interventions ranged from physical postures alone to integrated mind–body practices incorporating breathing exercises and meditation, while exercise programs varied substantially in duration and training prescription. These factors may have contributed to variability in treatment effects. Furthermore, only 13 studies were eligible for the preliminary meta-analysis, and only six studies could be incorporated into the final network because of the requirement for a connected treatment network. Consequently, more advanced analyses, such as subgroup analysis and meta-regression, were not feasible. Therefore, the present findings should be viewed as preliminary evidence intended to inform future research rather than provide definitive conclusions regarding the comparative effectiveness of exercise modalities for glycemic control in adults with type 2 diabetes mellitus.

## 5. Limitations of the Study

Several limitations of this study should be acknowledged. First, although 29 studies were included in the systematic review, only six trials were eligible for the final network meta-analysis. The sparse treatment network, limited number of interventions, and absence of closed-loop structures reduced statistical power and prevented formal inconsistency testing, node-splitting analyses, and robust assessment of publication bias.

Second, substantial clinical and methodological heterogeneity existed across studies, including variations in exercise intensity, intervention duration (10–52 weeks), participant characteristics, and intervention protocols. In addition, baseline physical activity levels, dietary intake, age at T2DM onset, and disease duration were not consistently reported, limiting the ability to perform subgroup analyses and fully evaluate potential effect modifiers.

Third, the comparison between HIIT and yoga was based entirely on indirect evidence through a common comparator. Therefore, the estimated treatment effects, SUCRA rankings, and ranking probabilities should be interpreted cautiously as exploratory findings rather than definitive evidence of superiority, particularly because the indirect comparison was not statistically significant (*p* = 0.766).

Fourth, the participant eligibility criterion was modified from the original PROSPERO-registered protocol. The initial protocol specified sedentary individuals with T2DM; however, this restriction was removed because sedentary status was inconsistently defined and reported across studies. Although this modification improved the comprehensiveness of the evidence base and did not alter the primary research question, it represents a protocol deviation and may have introduced selection bias.

Finally, the exclusion of non-English publications may have introduced language bias. This restriction was necessary because of limited access to professional translation resources required for accurate data extraction and synthesis.

Therefore, larger, well-designed head-to-head randomized controlled trials with standardized reporting of participant characteristics and intervention protocols are needed to confirm the comparative effectiveness of different exercise modalities for glycemic control in T2DM.

## 6. Conclusions

In conclusion, this pilot network meta-analysis suggests that both high-intensity interval training and yoga may improve glycemic control in patients with type 2 diabetes mellitus compared with active control. However, the treatment network was sparse, and the comparison between yoga and HIIT relied entirely on indirect evidence. Although yoga had the highest probability ranking, no statistically significant difference was observed between the two interventions. Therefore, the findings should be considered exploratory and hypothesis-generating rather than definitive.

Further well-designed head-to-head randomized controlled trials are needed to establish the comparative effectiveness of different exercise modalities for glycemic management in patients with type 2 diabetes mellitus.

## Figures and Tables

**Table 1:** Characteristics of included articles in the preliminary meta-analysis.

No.	Author/Year/Country	Design/Study Population	Intervention Group/Mean Age	Exercise Dose (Duration and Frequency)	Intensity	Monitoring of Exercise Intensity	Sample Size	Outcome (HbA1c) Changes		Main Findings
1	Abdelbasset et al. (2020)/Egypt [20]	RCT/T2DM	HIIT (Cycling Ergometer) Age (54.4 ± 5.8)	8 weeks: 3 times/week 40 min/session	80–85% VO_2_max (intense effort) 50% VO_2_max (light cycling)	VO_2_max	16	Baseline	After 8 weeks	Both the high-intensity (HII) and moderate-intensity (MIC) exercise groups showed reduced liver fat and belly fat. There was no clear difference between HII and MIC in their effects.
								6.6 ± 0.4	6.2 ± 0.3	
			Control Age (55.2 ± 4.3)	Receive medical treatment without exercise intervention			16	6.7 ± 0.6	6.5 ± 0.5	
2	Ahmad et al. (2023), Egypt [21]	Randomized Control Parallel/Women with T2DM	low-volume HIIT (Treadmill exercise) Age (42.96 ± 5.87)	12 weeks: 3 sessions/week, 19 min/session	Warm-up: 65–70% HRpeak Interval: 85–90% HRpeak Recovery: 65–75% Hrpeak	HR	24	Baseline	After 12 weeks	Both low- and high-volume HIIT showed improvements in TC, HDL, SBP, DBP, BMI, WC, and waist-to-hip ratio (*p* < 0.05). The high-volume HIIT group showed more significant improvements in HbA1c, FBG, 2 h PPBG, TG, LDL, (*p* < 0.05)
								8.15 ± 0.52	7.12 ± 0.49	
			High-volume HIIT (treadmill exercise) Age (43.29 ± 6.20)	12 weeks: 3 sessions/week, 16 min/session	Warm-up: 65–70% HRpeak Interval: 85–90% HRpeak Recovery: 65–75% Hrpeak		24	8.15 ± 0.56	6.65 ± 0.17	
			Usual care (non-exercising control group Age (42.46 ± 5.57)				24	8.14 ± 0.55	8.19 ± 0.50	
3	Cassidy et al. (2019)/UK [29]	RCT/T2DM	HIIT (Cycling Ergometer) Age (60 ± 3)	12 weeks: 3 sessions/week, 30–40 min/session	RPE 16–17 during intervals	Borg RPE scale	11	Baseline	12 weeks	HIIT group improved HbA1c (from 7.13% to 6.87%), while the control group worsened (from 7.18% to 7.36%), *p* = 0.03.
								7.13 ± 0.31	6.87 ± 0.29	
			Control Age (59 ± 3)	Usual care	Maintain their normal routine and do not change their medication, physical activity, diet or body weight.		11	7.18 ± 0.17	1.36 ± 0.21	
4	* Findikoglu et al. (2023)/Turkey [36]	a single-blinded, 3-arm, randomized, controlled prospective study/Individuals with T2DM for less than 10 years but more than 1 year	HIIT (Electromagnetic bicycle) Age (57.5 ± 7.82)	12 weeks: 3 sessions/week, 24 min in week (1–4) with 8 cycles, 36 min in week (5–8) with 12 cycles, and 48 min in week (9–12) with 16 cycles.	high-intensity (90% VO_2_peak for 60 s) low-intensity (30% VO_2_peak for 120 s)	VO_2_max	20	Baseline	after 12 weeks	Both HIIT and MICT improved VO_2_peak and HbA1c after 12 weeks of training. Only MICT caused additional improvements in cardiovascular responses, anthropometric measures, and abdominal fat compared to baseline (*p* < 0.05).
								6.9 ± 0.68	6.59 ± 0.49	
			Control Age (55.75 ± 8.56)	Doing simple static stretches of major muscle groups			20	6.99 ± 0.66	6.76 ± 0.66	
5	* Gupta et al. (2020)/India [25]	RCT/Individuals with T2DM	Yoga (asanas, kriyas, pranayama, and meditation) Age (50.6 ± 8.5)	16 weeks: Training phases: 3 sessions/week (weeks 1–2); Supervision phase: 2 sessions/week (weeks 3–4); Maintenance phase: 1 session/month (months 2–4). One Session/45 min.			40	Baseline	After 16 weeks	An HbA1c drop of ≥0.5% was observed in 44.7% of YBEP participants, while 37.5% in usual care. ≥75% of attending YBEP showed a 0.3% drop, compared to 0.1% for lower attendance.
								8.53 ± 0.71	8.31 ± 1.32	
			Control Age (50.6 ± 8.5)	usual care (Dietary counseling plus 30 min walking (5–6 km/h), ≥5 days/week.)			41	8.39 ± 0.65	8.38 ± 1.37	
6	Zheng et al. (2019)/China [18]	RCT/T2DM	high-intensity Progressive Resistance Training (using non-conventional equipment) Age (65.66 ± 8.58)	24 weeks: 1 session/week, 5–10 min/session Four isometric exercises (chest press, leg press, core pull, vertical lift) are supervised by a qualified trainer	Isometric contractions performed against resistance scaled to body weight, with progressive load increases.	Multiples of body weight (MOBs) generated during the isometric contractions	165	Baseline	After 24 weeks	There were no significant changes in HbA1c between the control and PRT groups overall. The intervention group had significant improvements in HDL and LDL
								6.83 ± 1.31	6.75 ± 0.93	
			Control Age (66.72 ± 6.68)	Participants maintained their usual medical care, dietary habits, and lifestyle.			100	6.92 ± 1.26	6.85 ± 1.17	
7	* Hirosaki et al. (2023)/Japan [37]	RCT/People with T2DM	Laughter Yoga Program (warm-up, deep breathing, laughter exercises, and calming activities) Age (71.8 ± 6.4))	12 weeks: Mini-lecture (30 min) + laughter yoga (60 min); 1 session/week (weeks 1–4), then 1 session/2 weeks (weeks 5–12).			21	Baseline	After 12 weeks	Laughter yoga group had a significant HbA1c reduction (−0.31%, 95% CI: −0.54 to −0.09) with increased positive affect scores (+0.62, 95% CI: 0.003 to 1.23).
								7.07 ± 0.7	6.82 ± 0.6	
			Control Age (70.6 ± 8.2)	Oral hypoglycemic medications, physician advice, and physical activity guidance under Japan’s *Treatment Guide for Diabetes*			21	7.19 ± 0.7	7.26 ± 0.7	
8	Hwang et al. (2019)/USA [39]	RCT/T2DM	HIIT (Cycling Ergometer) Age (65 ± 2)	8 weeks: 4 sessions/week, 40 min/session	90% HRpeak (4 × 4 min intervals) 70% HRpeak (3 × 3 min recovery)	HR	23	Baseline	After 8 weeks	No improvement has been found in glycemic control, lipid profile, or blood pressure. However, both intervention groups improved in aerobic fitness.
								7.1 ± 0.3	6.8 ± 0.2	
			Control Age (61 ± 2)	Followed regular physical activity, dieting, and meditation			16	7.4 ± 0.4	7.5 ± 0.4	
9	* Li et al. (2022)/China [31]	parallel randomized controlled clinical trial/T2DM patients	HIIT (Swedish Monark power bike) Age (38 ± 6)	12 weeks: 5 sessions/week, 30 min/session	80–95% HRmax/VO_2_peak	HR	13	Baseline	After 12 weeks	The MICT group showed significant weight loss (difference = 3.52, *p* < 0.01) and improved FBG (*p* < 0.05). BMI significantly decreased within groups (*p* < 0.01), though not between groups. HbA1c levels improved significantly over time (*p* < 0.01), but not between groups. SBP improved significantly in both HIIT and MICT groups compared to the control (*p* < 0.05).
								7.18 ± 0.50	6.79 ± 0.41	
			Control Age (40 ± 7)	Received standard counseling on conventional T2DM exercise guidelines			12	7.06 ± 0.38	7.09 ± 0.33	
10	Ma et al. (2024)/China [34]	Single-blinded RCT/People diagnosed with T2DM	RT (using dumbbells, resistance bands, and kettlebells) Age (66.65 ± 4.94)	24 weeks: 3 times/week, 50 min/time	40–50% of 1-RM during initial training, progressively increasing by 5–10% up to 60–70% of 1RM.	% of 1-RM	31	Baseline	24 weeks	Fasting Plasma Glucose (FPG), HbA1c, blood lipids, diastolic blood pressure, body composition, and muscle performance significantly improved in both exercise groups compared to the control group and their own baseline (*p* < 0.05).
								7.80 ± 0.93	7.11 ± 0.75	
			blood flow restrictive resistance exercise group (using KAATSU Air Bands) Age (66.41 ± 4.97)	24 weeks: 3 times/week, 50 min/time	20–30% of one-repetition maximum (1RM) combined with blood flow restriction pressure sufficient to partially restrict arterial inflow.	% of 1-RM for load resistance and limb occlusion pressure (LOP) for BFR	34	7.75 ± 0.97	7.24 ± 0.85	
			Control Age (65.55 ± 4.41)	Usual care (followed a diabetes management program but received no formal training and continued their usual self-selected diet and exercise habits.)			33	7.98 ± 0.96	7.93 ± 0.75	
11	* Viswanathan et al. (2021)/India [28]	RCT/T2DM	Yoga (loosening exercises, asanas, pranayama, and relaxation techniques, along with diabetes education) Age (50.8 ± 8.3)	12 weeks: 5 days/week, 50 min/day			150	Baseline	After 12 weeks	Yoga group lowers BMI, blood sugar, HbA1c, cholesterol, and inflammation markers.
								7.5 ± 0.5	7.2 ± 0.9	
			Non-Yoga Age (52.8 ± 7.0)	Simple physical exercises for 50 min per session, 5 days per week.			150	7.5 ± 0.6	7.6 ± 1.1	
12	* Way et al. (2020)/Australia [42]	RCT/T2DM	HIIT (Cycling Ergometer) Age (56.9 ± 2.1)	12 weeks: 3 sessions/week, 19 min/session	Each session with 4 min of high-intensity cycling at 90% VO_2_peak	% of VO_2_peak	12	Baseline	After 12 weeks	After the intervention of HIIT and MICT, significant improvements were found in VO_2_peak (*p* < 0.01), HbA1c (*p* = 0.03), systolic blood pressure (*p*< 0.01), and waist size (*p* = 0.03).
								7.1 ± 1.5	6.8 ± 0.9	
			Control Age (51.9 ± 1.4)	Sham Exercise Placebo (Stretching and core exercises every 2 weeks; ≤30 min/session with 5 min light cycling (20 W) warm-up and cool-down.)			11	7.6 ± 0.5	8.0 ± 0.5	
13	Yamamoto et al. (2021)/Japan [38]	Prospective RCT/T2DM	Resistance exercise (using an elastic band) Age (73.2 ± 2.6)	48 weeks of daily exercise: one time/15 min	Bodyweight exercises combined with progressive elastic band resistance (TBB-1 to TBB levels) representing increasing resistance levels.	Daily exercise logs (adherence tracking).	18	Baseline	After 48 weeks	No changes in physical function, muscle mass, or cognitive function in any group. The leucine supplement brought no extra benefits for muscle strength or mass.
								7.4 ± 0.9	7.3 ± 0.8	
			Control Age (73.3 ± 2.5)	Maintain daily activities			17	7.0 ± 0.7	6.8 ± 0.6	

* articles included in NMA.

**Table 2:** Results of the random-effects network meta-analysis consistency model for HbA1c Change.

Comparison	Mean Difference (MD)	95% CI	*p*-Value
HIIT vs. Active control	−0.322	−0.559, −0.084	0.008
Yoga vs. Active control	−0.366	−0.534 to −0.198	<0.001
HIIT vs. Yoga (indirect)	−0.044	−0.335 to 0.247	0.766

Heterogeneity parameters: between-study standard deviation (τ): 2.91 × 10^−12^; assumed between-study correlation: 0.50.

**Table 3:** League table of network meta-analysis for HbA1c change.

Treatment	Active Control	HIIT	Yoga
Active control	—	0.322 (0.084 to 0.559)	0.366 (0.198 to 0.534)
HIIT	−0.322 (−0.559 to −0.084)	—	−0.044 (−0.335 to 0.247)
Yoga	−0.366 (−0.534 to −0.198)	0.044 (−0.247 to 0.335)	—

Values are presented as mean differences (MDs) with 95% confidence intervals. Negative values indicate greater reductions in HbA1c.

**Table 4:** Ranking probabilities and SUCRA values for interventions.

Rank	Intervention	Probability of Being Best	Probability of Being Second	Probability of Being Third	SUCRA
1	Yoga	1.0	0.0	0.0	1.0
2	HIIT	0.0	1.0	0.0	0.5
3	Active control	0.0	0.0	1.0	0.0

SUCRA: surface under the cumulative ranking curve.

## Data Availability

No new data were created or analyzed in this study.

## Supplementary Materials

The following supporting information can be downloaded at: https://www.mdpi.com/article/10.3390/healthcare14121703/s1. Supplementary File S1: Date S1: Search term and search string; Table S1: JBI quality assessment of qualitative articles, Table S2: Characteristics of all the included articles in qualitative synthesis; Table S3: Study effect and comparison-adjusted funnel plot; Table S4: Arm-level data of studies included in NMA. Supplementary File S2: PRISMA checklist. Ref. [53] is cited in File S2.
