# Supplementary material for: Yoga and High-Intensity Interval Training Show Comparable Effects on HbA1c in Type 2 Diabetes: A Systematic Review and Preliminary Pilot Network Meta-Analysis in Adult Populations
Source: Healthcare (Basel). 2026 Jun 15;14(12):1703. doi: 10.3390/healthcare14121703 (PMC13299578; doi:10.3390/healthcare14121703)
Supplement: Supplementary file 1 [file healthcare-14-01703-s001.zip › Supplementary file S1.pdf]

Supplementary Data S1: Search Term and Search String

PICO criteria:
The population (P) comprised adults aged ≥18 years with type 2 diabetes mellitus (T2DM), as defined in the included studies. The intervention (I) included any form of physical exercise, such as high-intensity interval training (HIIT), resistance training (RT), yoga, or Tai Chi. HIIT was defined as exercise consisting of repeated bouts of high-intensity activity (typically >80% of maximal heart rate [HRmax]) interspersed with periods of low-intensity recovery or rest. Yoga and Tai Chi were defined as mind–body practices integrating physical postures and breathing techniques. The comparison (C) group received usual care, defined as standard medical treatment or advice without a structured exercise intervention, or sham exercise, defined as very low-intensity activities (e.g., stretching or core exercises) intended as a placebo. The outcome (O) was the change in glycated haemoglobin (HbA1c).

Database-specific search strategies were developed using a combination of controlled vocabulary (e.g., MeSH terms where applicable) and free-text keywords.

The core search terms based on PICO criteria included:

"type 2 diabetes mellitus," "T2DM," "hyperglycemia," "physical exercise," "aerobic exercise," "resistance training," "walking," "running," "high-intensity interval training," "HIIT," "yoga," "tai chi," "HbA1c," "glycemic control," "postprandial glucose," "fasting blood glucose," "insulin sensitivity," "body mass index," "body weight," "blood pressure," and "waist circumference."

These terms were adapted for each database and combined using Boolean operators "**AND**" and "**OR**."

An example of the PubMed search strategy was as follows:

("Type 2 Diabetes Mellitus" OR "T2DM" OR "hyperglycemia") AND ("high-intensity interval training" OR "HIIT" OR "aerobic exercise" OR "resistance training" OR "yoga" OR "tai chi") AND ("HbA1c" OR "glycemic control" OR "fasting blood glucose" OR "postprandial glucose").

Similar search logic was applied and appropriately modified for CINAHL, MEDLINE, the Cochrane Library, and ProQuest according to their indexing systems and database-specific syntax requirements. Full search strategies for each database are provided in the Supplementary Materials to ensure transparency and reproducibility

Supplementary Table S1: JBI Quality Assessment of Randomized Controlled Trials (RCTs)

		True randomization	Allocation concealment	Baseline similarity	Participant blinding	Provider blinding	Outcome assessor blinding	Identical treatment except intervention	Complete follow-up	Intention-to-treat analysis	Same outcome measurement	Reliable outcome measures	Appropriate statistical analysis	Appropriate trial design and handling deviations	Result	Risk
1	#Abdelbasset et al (2019) [20]	Y	Y	Y	N	N	Y	Y	Y	Y	Y	Y	Y	Y	84.62	Low
2	#Ahmad et al. (2023) [21]	Y	Y	Y	N	N	N	Y	Y	Y	Y	Y	Y	Y	76.92	Low
3	Ali et al. (2024) [19]	Y	Y	Y	N	N	U	Y	Y	Y	Y	Y	Y	Y	76.92	Low
4	Al-Rawaf et al (2023) [22]	N	N	U	N	N	N	Y	Y	N	Y	Y	Y	U	38.46	High
5	Amaravadi et al(2024) [23]	Y	Y	Y	N	N	U	Y	Y	Y	Y	Y	Y	Y	76.92	Low
6	#Cassidy et al (2019) [29]	Y	Y	Y	N	N	U	Y	Y	Y	Y	Y	Y	Y	76.92	Low
7	Chan et al (2018) [13]	Y	Y	Y	N	N	Y	Y	Y	Y	Y	Y	Y	Y	84.62	Low
8	Chattopadhyay et al (2023) [24]	Y	Y	Y	N	N	Y	Y	Y	Y	Y	Y	Y	Y	84.62	Low
9	#* Findikoglu et al (2023) [36]	Y	Y	Y	N	N	Y	Y	Y	Y	Y	Y	Y	Y	84.62	Low
10	#* Gupta et al (2020) [25]	Y	Y	Y	N	N	Y	Y	Y	Y	Y	Y	Y	Y	84.62	Low
11	#Hangping et al (2019) [18]	Y	Y	Y	N	N	Y	Y	Y	Y	Y	Y	Y	Y	84.62	Low
12	#* Hirosaki et al (2023) [37]	Y	Y	Y	N	N	Y	Y	Y	Y	Y	Y	Y	Y	84.62	Low
13	#Hwang et al (2019) [39]	Y	U	Y	N	N	Y	Y	Y	Y	Y	Y	Y	Y	76.92	Low
14	Leung et al (2019) [30]	Y	Y	Y	N	N	Y	Y	Y	N	Y	Y	Y	Y	76.92	Low
15	Li et al (2020) [32]	Y	Y	Y	N	N	Y	Y	Y	Y	Y	Y	Y	Y	84.62	Low
16	#* Li et al (2022) [31]	Y	Y	Y	N	N	U	Y	Y	Y	Y	Y	Y	Y	76.92	Low
17	Liu et al (2024) [33]	Y	U	Y	N	N	Y	Y	Y	Y	Y	Y	Y	Y	76.92	Low
18	#Ma et al (2024) [34]	Y	U	Y	N	N	Y	Y	Y	U	Y	Y	Y	Y	69.23	Moderate
19	Magalhães et al (2018) [40]	Y	N	Y	N	N	U	Y	Y	Y	Y	Y	Y	Y	69.23	Moderate
20	Mir et al (2020) [16]	Y	U	Y	N	N	U	Y	Y	U	Y	Y	Y	Y	61.54	Moderate
21	Nazari et al (2023) [17]	Y	U	Y	N	N	U	Y	Y	U	Y	Y	Y	Y	61.54	Moderate

22	Qian et al (2024) [35]	Y	U	Y	N	N	U	Y	Y	U	Y	Y	Y	Y	61.54	Moderate
23	Ranasinghe et al (2021) [41]	Y	U	Y	N	N	Y	Y	Y	N	Y	Y	Y	Y	69.23	Moderate
24	Ranga et al (2021) [26]	U	N	Y	N	N	N	Y	U	U	Y	U	Y	N	30.77	High
25	Sivapuram et al (2020 [27]	Y	N	Y	N	N	U	Y	Y	Y	Y	Y	Y	Y	69.23	Moderate
26	Sudarsono et al (2019) [43]	Y	U	Y	N	N	Y	Y	Y	U	Y	Y	Y	Y	69.23	Moderate
27	#*Viswanathan et al (2021) [28]	Y	U	Y	N	N	U	Y	Y	N	Y	Y	Y	Y	61.54	Moderate
28	#*Way et al., (2020) [42]	Y	Y	Y	N	N	U	Y	Y	Y	Y	Y	Y	Y	76.92	Low
29	#Yamamoto et al (2021) [38]	Y	U	Y	N	N	U	Y	Y	Y	Y	Y	Y	Y	69.23	Moderate

Note:* # *Articles included in preliminary meta-analysis,* * *Articles included in NMA.,* #* *Articles included in both

Supplementary Table S2: Characteristics of the included articles

No	Author/Year/ Country	Design/Study population	Intervention Group/Mean age	Type of intervention	Exercise Dosage (Frequency and duration)	Intensity	Monitoring of exercise intensity	Sample Size	Outcome (HbA1c) changes		Main Findings	Reason for exclusion in the NMA
									Baseline	after 8 weeks		
1	#Abdelbasset et al (2019)/ Egypt [20]	RCT/T2DM	HIIT Age (54.4±5.8)	High-intensity aerobic exercise performed on a cycle Ergometer	8 weeks: 3 times/week, one time/ 40 minutes	80–85% VO_2max (intense effort) 50% VO_2max (light cycling)	VO_2max	16	6.6±0.4	6.2±0.3	Both the high-intensity (HII) and moderate-intensity (MIC) exercise groups showed reduced liver fat and belly fat. There was no clear difference between HII and MIC in their effects.	
			MIIT Age (54.9±4.7)	Moderate intensity aerobic exercise performed on a cycle Ergometer	3 times/week for 8 weeks, one time/ 40-50 minutes	Continuous cycling at 60–70% of maximum heart rate (HRmax)		15	6.4±0.5	6.0±0.4		
			Control Age (55.2±4.3)	Receive medical treatment without exercise intervention				16	6.7±0.6	6.5±0.5		
									Baseline	After 12 weeks		
2	#Ahmad et al. (2023), Egypt [21]	Randomized Control Parallel/ Women with T2DM	low-volume HIIT Age (42.96 ± 5.87)	Modified Bruce treadmill test at baseline and 12 weeks under standardized conditions (usual medication and	12 weeks: 3 sessions/week, 19 minutes/session	Warm-up: 65–70% HRpeak Interval: 85–90% HRpeak Recovery: 65–75% Hrpeak	HR	24	8.15 ± 0.52	7.12 ± 0.49	Both low- and high-volume HIIT showed improvements in TC,	

			high-volume HIIT Age (43.29 ± 6.20)	hydration maintained).	12 weeks: 3 sessions/week, 16 minutes/session	Warm-up: 65–70% HRpeak Interval: 85–90% HRpeak Recovery: 65–75% Hrpeak		24	8.15 ± 0.56	6.65 ± 0.17	HDL, SBP, DBP, BMI, WC, and waist-to-hip ratio (p <0.05). The high-volume HIIT group showed more significant improvements in HbA1c, FBG, 2-hr PPBG, TG, LDL, (p < 0.05)	
			Non-exercising control group Age (42.46 ± 5.57)	Usual care				24	8.14 ± 0.55	8.19 ± 0.50		
3	Ali et al. (2024)/UAE [19]	RCT/ adults with T2DM	Structured exercise (Aerobic and strengthening) Age (53.04±9.02)	Home-based aerobic and strengthening exercise with diabetes nutrition counselling and dietary modification for 6 weeks.	48 weeks: Light intensity (15-min session); light-to-moderate intensity (30- and 60-min sessions).	Light to light-to-moderate intensity.	Not reported	163	Baseline<br>7.45 ± 0.11	After 48 weeks<br>7.44 ± 0.13	No changes in HbA1c but BMI and daily caloric intake were significantly decreased in the intervention compared to the control group by 1.18 kg/m2 (95% CI: -1.78 − -0.60) and 246 kcal (95% CI: -419.52 − -77.21)	Missing or non-extractable data<br>Inconsistent outcome reporting<br>Insufficient network connectivity
			Control Age (54.92±9.96)	Usual care	Receiving diabetes management education as part of the diabetes management from physicians and nurses.			219	7.81 ± 0.11	7.64 ± 0.11		
4	Al-Rawaf (2023)/Saudi Arabia [22]	RCT/T2DM	HIIT Age (: 46.1 ± 3.1)	Using an electronic treadmill	12 weeks: 3 times per week, one session/40 mins 10-minute warm-up (at 50% HRmax), four 4-minute high-intensity intervals	3 sessions/week for 12 weeks, 40 min/session; treadmill-based HIIT with warm-up,	HRmax and Borg RPE scale.	30	Baseline<br>7.4 ± 1.6	after 12 weeks<br>5.2 ± 2.5	HIIT intervention led to significant improvements in FBG, HbA1c, insulin, C-	Missing or non-extractable data

					(at 80–85% HRmax) with 3-minute active recovery periods (at 70% HRmax)	4 × 4-min intervals, recovery, and cool-down.					peptide, and HOMA-IR in both control and intervention groups	Inconsistent outcome reporting<br>Insufficient network connectivity
			Control Age (46.3 ± 2.8)	Usual daily activities	Receive no specific exercise intervention			20	4.6 ± 0.45	3.2 ± 0.65		
5	Amaravadi et al. (2024)/India [23]	RCT/individuals with T2DM							Baseline	after 12 weeks		Missing or non-extractable data<br>Inconsistent outcome reporting<br>Insufficient network connectivity
			Structured exercise program (Aerobic and strengthening) Age (56.05 ± 8.77)	Aerobic (Brisk walking and on the treadmill) and resistance exercise program	12 weeks: Aerobic (3-5time/week, 15-20 minutes to 30-45 minutes/session)<br>12 weeks: Resistance exercise (3-5 times/week, 10-20 minutes to 30-60 minutes/session	Moderate-intensity walking corresponding to a Borg RPE of 12–13 (6–20 scale) or 4–6 (0–10 scale) with progression adjusted at week 6 based on 6-minute walk distance (6MWD).	Borg RPE scale + 6-minute walk test (6MWD)	75	8.06 ± 0.73	8.45 ± 0.80	Homa-IR (F (1, 144) = 89.29, p < 0.001); Fasting insulin (FI) (F (1, 144) = 129.10, p < 0.001); Fasting blood sugar (FBS) (F (1, 144) = 12.193, p< 0.001); Postprandial blood sugar (PPBS) (F (1, 144) = 53.015, p< 0.001); glycated haemoglobin (HbA1c) (F (1, 144) = 80.050, p < 0.001)	
			Control Age (53.90 ± 10.20)	standard care	standard hospital care based on medical recommendations			71	8.11± 1.27	7.52 ± 1.05		
6	#Cassidy et al (2019)/UK [29]	RCT/T2DM	HIIT Age (60±3)	Using a cycle ergometer, Participants were instructed not to change their diet, medication, or usual physical	12 weeks: 3 sessions per week, each session/30-40 minutes with first session supervised,	RPE 16–17 during intervals	Borg RPE scale	11	Baseline<br>7.13 ± 0.31	12 weeks<br>6.87 ± 0.29	HIIT group improved HbA1c (from 7.13% to 6.87%), while the control	

				activity during the 12-week period.	remaining self-guided.						group worsened (from 7.18% to 7.36%), p = 0.03.	
			Control Age (59±3)	Usual care	Maintain their normal routine and do not change their medication, physical activity, diet or body weight.			11	7.18 ± 0.17	7.36 ± 0.21		
7	Chan et al. (2018)/ China [13]	RCT/Patients with diabetes, hypertension and dyslipidaemia					HR		Baseline	after 12 weeks	Tai Chi and brisk walking lower SBP, DBP, fasting blood sugar, HbA1c, reduce stress and improve mental health.	Missing or non-extractable data<br>Inconsistent outcome reporting<br>Insufficient network connectivity
			Tai Chi Age (64.70 ± 7.59)	24-form Yang Style Tai Chi (structured group-based exercise)	12 weeks: 2 times/week, 60 minutes/session, led by a qualified Tai Chi Master, with home practice 30 min/day, 5 days/week			82	6.66±1.17	6.38±0.90		
			Bris Walking Age (63.22 ± 11.11)	Brisk walking (individual aerobic exercise)	12 weeks, 5 days/ week, 30 minutes/day, with walking speed: 5–6 km/h	Moderate intensity (5–6 km/h; age-adjusted HR).		82	7.10±1.61	6.66±1.02		
			Control Age (65.13 ± 10.22)	Participants continued their usual activities				82	6.87±1.25	6.70±1.30		
8	Chattopadhyay et al (2023)/India [24]	A multicenter, two-arm, parallel-group, feasibility RCT/ individuals with T2DM	Yoga Age (41.3 ±7.4)	Yoga-Based Physical Activity	24 weeks: 3-5 times/week. 27 group Yoga sessions and self-practice at home using the program booklet and a video.27 Types of Yoga-based physical activity, including Shithilikarana Vyayama			33	Baseline	after 24 weeks	BMI was significantly lower in the intervention group (ß= -0.56; 95% CI: -1.00 to -0.11). Fasting blood glucose, HbA1c, and waist circumferen	Missing or non-extractable data<br>Inconsistent outcome reporting<br>Insufficient network connectivity
									5.9 ±0.3	5.6 ±0.4		

					(loosening exercises), Surya Namaskar (sun salutation exercises), Asana (Yogic poses), Pranayama (breathing practices), and Dhyana (meditation) and relaxation practices.						ce were also lower, but not significantly.	
			Control Age (42.8 ± 8.0)	Routine lifestyle care	Provision of leaflet and health-related advice			32	5.9 ±0.4	5.6 ± 0.5		
9	#*Findikoglu et al (2023)/Turkey [36]	A single-blinded, 3-arm, randomized, controlled prospective study/Individuals with T2DM for less than 10 years but more than 1 year	HIIT Age (57.5 ± 7.82)	Training with an electromagnetic bicycle.	12 weeks: 3 sessions/week, 24 minutes in week (1-4) with 8 cycles, 36 minutes in week (5-8) with 12 cycles, and 48 minutes in week (9-12) with 16 cycles.	high-intensity (90% VO_2peak for 60s) low-intensity (30% VO_2peak for 120s)	VO_2max	20	Baseline	after 12 weeks	Both HIIT and MICT improved VO2peak and HbA1c after 12 weeks of training. Only MICT caused additional improvements in cardiovascular responses, anthropometric	
									6.9 ± 0.68	6.59 ± 0.49		

			MIIT Age (55.42 ± 8.12)		12 weeks: 3 sessions/week, 24 minutes in week (1-4) with 8 cycles, 36 minutes in week (5-8) with 12 cycles, and 48 minutes in week (9-12) with 16 cycles.	Steady cycling at 50% VO_2peak		20	6.98 ± 1.27	6.47 ± 0.79	measures, and abdominal fat compared to baseline ($p < 0.05$).	
			Control Age (55.75 ± 8.56)	Simple static stretches of	Simple static stretches of major muscle groups at home for 12 weeks			20	6.99 ± 0.66	6.76 ± 0.66		
10	#*Gupta et al (2020)/India [25]	RCT/Individuals with T2DM							Baseline	after 16 weeks	An HbA1c drop of ≥0.5% was observed in 44.7% of YBEP participants, while 37.5% in usual care. ≥75% of of attending YBEP occurred a 0.3% drop, compared to 0.1% for lower attendance.	
			Yoga Age (50.6 ±8.5)	Asanas (yoga postures), riyas (cleansing practices), pranayama (breathing exercises), and meditation.	16 weeks: Training phases: 3 sessions/week (weeks 1- 2); Supervision phase: 2 sessions/week (weeks 3–4); Maintenance phase: 1 session/month (months 2–4). One Session/ 45 min.			40	8.53±0.71	8.31 ±1.32		
			Control Age (50.6 ±8.5)	Usual care	Dietary counselling plus 30 min walking (5–6 km/h), ≥5 days/week.)			41	8.39±0.65	8.38 ± 1.37		
11	#Hangping (2019)/ China [18]	RCT/T2DM	High-intensity Progressive Resistance Training	Perform strength training using non-conventional equipment	24 weeks: 1 session/week, 5-10 minutes/session Four isometric		Multiples of body weight (MOBs)	165	Baseline	After 24 weeks	There were no significant changes in HbA1c	
									6.83 ± 1.31	6.75 ± 0.93		

			(PRT) Age (65.66 ± 8.58)		exercises (chest press, leg press, core pull, vertical lift) are supervised by a qualified trainer		generated during the isometric contractions				between the control and PRT groups overall. The intervention group had significant improvements in HDL and LDL	
			Control Age (66.72 ± 6.68)	Participants maintained their usual medical care, dietary habits, and lifestyle.	All participants were instructed to maintain their usual medical care, nutritional habits and other current lifestyle patterns			100	6.92 ± 1.26	6.85 ± 1.17		
12	#*Hirosaki et al (2023)/Japan [37]	RCT/People with T2DM	Laughter Yoga Program Age (71.8 ± 6.4))	Laughter Yoga Program (warm-up, deep breathing, laughter exercise-es, and calming activities)	12 weeks: Mini-lecture (30 min) + laughter yoga (60 min); 1 session/week (weeks 1–4), then 1 session/2 weeks (weeks 5–12).			21	Baseline	After 12 weeks	laughter yoga group had a significant HbA1c reduction (−0.31%, 95% CI: −0.54 to −0.09) with increased positive affect scores (+0.62, 95% CI: 0.003 to 1.23).	
									7.07±0.7	6.82 ± 0.6		
			Control Age (70.6 ±8.2)	Standard therapy for diabetes	Taking oral hypoglycemic medications, Receiving advice from the doctor and physical activity under the "Treatment Guide for Diabetes" in Japan			21	7.19 ± 0.7	7.26 ± 0.7		

13	#Hwang et al. (2019)/USA [39]	RCT/T2DM	HIIT Age (65 ± 2)	Using ergometer	8 weeks: 4 sessions/week, 40 minutes/session	90% HRpeak (4 × 4-min intervals) 70% HRpeak (3 × 3-min recovery	HR	23	Baseline	After 8 weeks	No improvement has been found in glycemic control and lipid profile as well as blood pressure. However, both intervention groups improve in aerobic fitness.	
									7.1 ± 0.3	6.8 ± 0.2		
			MICT Age (62 ± 2)		8 weeks: 4 session/week, 47 minutes/session	70% HRpeak (continuous exercise, 32 min)		19	7.2 ± 0.3	7.0 ± 0.2		
			Control Age (61 ± 2)	Perform daily routine	Follow regular physical activity, dieting and meditation			16	7.4 ± 0.4	7.5 ± 0.4		
14	Leung et al. (2019)/China [30]	RCT/Patients with metabolic syndrome	Tai Chi Age (62.19 ±5.93)	24-form Yang-style Tai Chi	12 weeks: twice/week, 60 minutes/session led by a qualified Tai Chi master. Home practice 3 times/ week, one time/30 minutes			18	Baseline	After 12 weeks	The Tai Chi group lowers SBP and increases perceived mental health, while there is no difference found in the control group.	Missing or non-extractable data<br>Inconsistent outcome reporting<br>Insufficient network connectivity
									7.51 ±1.34	7.37 ±1.71		
			Control Age (65.52 ± 9.34)	Non-exercise recreational activities				17	7.57 ±0.82	7.51 ±1.16		
								34	Baseline	12 weeks		

15	Li et al. (2020)/China [32]	Single-blinded, randomized, controlled trial/Patients with T2DM	Qigong Age (59.71 ± 6.67)	Self-massage, physical and breathing exercise	12 weeks: One time/week for 60 minutes				7.99 ± 1.66	7.98±1.50	After 12 weeks, HbA1c and C-peptide levels improved (p = 0.010, p = 0.031). Tai chi significantly reduced C-peptide (p = 0.004). In qigong, longer T2DM related to smaller FPG changes while in tai chi, HbA1c changes were linked to waist-to-height ratio	Missing or non-extractable data<br>Inconsistent outcome reporting<br>Insufficient network connectivity
			Tai chi Age (61.71 ± 6.91)	Physical and breathing exercise	12 weeks: One time/week for 60 minutes			24	8.20 ± 2.46	8.65±1.86		
			Stretching Control Age (58.66 ± 10.89)	Stretching exercises (upper body, trunk, lower body) with controlled breathing and relaxation	5 sessions for the first week with 40 minutes/session. One session each for the 11 weeks.			29	7.63 ± 1.74	7.23±1.25		
16	#*Li et al. (2022)/China [31]	parallel randomized controlled clinical trial/T2DM patients					HR		Baseline	After 12 weeks	The MICT group showed significant weight loss (difference = 3.52, p<0.01) and improved FBG (p <0.05). BMI significantly decreased within groups (p < 0.01), though not between groups. HbA1c levels	
			HIIT Age (38 ± 6)	Swedish Monark power bike as a device for the exercise intervention	12 weeks: 5 sessions/week, 30 min/session	80–95% HRmax / VO_2peak		13	7.18 ± 0.50	6.79 ± 0.41		
			MICT Age (39 ± 5)		12 weeks: 5 sessions/week, 15 minutes/session	50%–70% HRmax / VO_2peak		12	7.02 ± 0.44	6.88 ± 0.40		
			Control Age (40 ± 7)	Received standard counseling on conventional T2DM exercise	Receive standard counseling on conventional T2DM exercise guidelines			12	7.06 ± 0.38	7.09 ± 0.33		

											improved significantly over time (p <0.01), but not between groups. SBP improved significantly in both HIIT and MICT groups compared to control (p <0.05).	
17	Liu et al. (2024)/China [33]	RCT/ People with T2DM					HR		Baseline	After 24 weeks	Taichi (with or without resistance band training) significantly improved insulin sensitivity and reduced fasting blood glucose, insulin, HbA1c, and harmful inflammation markers.	Missing or non-extractable data<br>Inconsistent outcome reporting<br>Insufficient network connectivity
			Tai Chi Training Age (60.6 ± 5.7)	Yang style Tai Chi exercise includes 24 movements. All participants were requested to maintain their daily routine, medications, and dietary habits during the study period.	24 weeks: 6 days/week, 60 minutes/session	Low-to-moderate (HR 95–105 bpm)		16	7.46±1.13	6.57±0.9		
			Tai Chin plus resistance band Training Age (61.4 ± 3.4)	Tai Chin with resistance training including shoulder flexion and abduction, triceps extension, biceps curl, squat, forward lunge, hip adduction/abduction, hip extension by using elastic bands	Tai Chi<br>24 weeks: 60 mins/session, 4 days/week.<br><br>Resistance Band<br>24 weeks: 60 mins/day<br>2 days/week for 24 weeks	40–50% 1RM, 2 sets of 8–12 reps		16	7.50±1.06	6.45±0.97		

			Control Age (61.3 ± 4.9)	Regular treatment	Regular treatment for diabetes care and were requested to maintain their lifestyle daily without doing exercise and to record the activity daily.			16	7.40±0.74	7.47±0.77		
18	#Ma et al. (2024)/China [34]	Single blined RCT/ People diagnosed with T2DM	RT Age (66.65 ± 4.94)	Participants used simple equipment (dumbbells, resistance bands, kettlebells) for home-based training targeting upper body, lower body, and core.	3 times/week One time 50 minutes	40–50% of 1RM during initial training, progressively increasing by 5–10% up to 60–70% of 1RM.	% of 1-RM	31	Baseline	24 weeks	Fasting Plasma Glucose (FPG), HbA1c, blood lipids, diastolic blood pressure, body composition, and muscle performance significantly improved in both exercise groups compared to the control group and their own baseline (P < 0.05).	
									7.80 ± 0.93	7.11 ± 0.75		
			Blood flow restrictive resistance exercise group (BFRE) Age (66.41 ± 4.97)	KAATSU Air Bands applied for blood flow restriction, with resistance training at 20–30% 1RM.	24 weeks: 3 times/week, 50 minutes/time	20–30% of one-repetition maximum (1RM) combined with blood flow re-striction pressure sufficient to partially restrict arterial inflow.	% of 1-RM for load resistance and limb occlusion pressure (LOP) for BFR	34	7.75 ± 0.97	7.24 ± 0.85		
			Control Age (65.55 ± 4.41)	Usual care	24 weeks: 3 times/week, 50 minutes/time			33	7.98 ± 0.96	7.93 ± 0.75		
19	Magalhães et al. (2018)/	RCT/ T2DM	MCT combined	HIIT and MCT do cycling, and	12 weeks: 3 sessions/week,	Continuous cycling at 40–	HR (Polar	25	Baseline	After 12 weeks	neither HIIT+RT	

	Portuguese [40]		with RT Age (59.7 ± 6.5)	RT includes seated row, lat pulldown, chest press, leg press, planks	one session/ about 45 minutes, supervised by an exercise physiologist	60% heart rate reserve (HRR) combined with full-body strength exercises.	T-31 heart rate bands)		53± 17.4	54± 14.8	nor MCT+RT significantly improved HbA1c. However, the MCT+RT group reduced body fat and improved cardiorespira tory fitness.	
			HIIT combined with RT Age (56.7 ±8.3)		12 weeks:3 sessions/week, about 33 minutes/session, supervised by an exercise physiologist	Weeks 1–4: Moderate-intensity continuous cycling at 40–60% HRR. Weeks 5–8: Interval training at 70–80% HRR with active recovery at 40–60% HRR (2 min work/1 min rest). Weeks 9–52: HIIT at 90% HRR with active recovery at 40–60% HRR (1 min work/1 min rest).		28	52.1± 9.6	52.8± 7.1		Missing or non-extractable data<br>Inconsistent outcome reporting<br>Insufficient network connectivity
			Control Age (59.0± 8.1)	No structured exercise sessions but participants were invited to an orientation session during which standard counselling regarding general PA				27	51.7± 11.7	54.8± 11.1		

				guidelines was provided								
20	Mir (2020)/Iran [16]	RCT/T2DM							Baseline	After 12 weeks	After HIIT and resistance training intervention, improvement found in SFRP5 levels, reduced WNT5A, HbA1c, insulin resistance, BMI, and body fat.	Missing or non-extractable data<br>Inconsistent outcome reporting<br>Insufficient network connectivity
			HIIT combined with RT Age (58.9 ± 3.54)	HIIT: Treadmill-based training<br><br>RT: Six resistance exercises (leg press, chest press, front thigh, lat pull-down, back thigh, and shoulder press)	12 weeks: 3 sessions/week, HIIT (21 min) + RT (20 min)	HIIT: 3 × 4-min intervals at 70–90% HRmax, with 3-min active recovery at 50–70% HRmax. Resistance training: 55–80% 1RM.	HR and %1RM	10	7.49 ± 0.86	6.97 ± 0.89		
			Control Age (57.7 ± 4.57)	Usual sedentary lifestyle maintained; medication changes reported to researchers.				9	7.13 ± 0.68	7.54 ± 0.70		
21	Nazari et al. (2023)/Iran [17]	RCT/T2DM							Baseline	After 12 weeks	HbA1c improved significantly in the CRT group (P = 0.01). Total cholesterol decreased in both CRT (P = 0.01) and jogging groups (P < 0.05)	Missing or non-extractable data<br>Inconsistent outcome reporting<br>Insufficient network connectivity
			Circuit resistance training (CRT) Age (50.20±4.89)	Machine-based resistance training including bench press, seated row, lat pull-down, biceps curl, leg extension, leg curl, leg press, and rowing.	12 weeks: 3 sessions/week, 60 minutes/session	Weeks 1–6: 40–50% of 1RM<br>Weeks 7–12: 50–65% of 1RM	%1RM	10	7.19±0.4	6.1±0.5		
			Jogging Age (52.60±5.33)	Walking and jogging	12 weeks: 3 times/week, 36 sessions in total, 25-50 minutes/ session	60% Targeted HR (early phase) to 75% Targeted HR (later phase)	Targeted heart rate (THR)	10	7.33±0.6	7.2±0.7		
			Control Age (51.30±5.63)	Usual care				10	7.03±0.4	7.0±0.4		
22		RCT/T2Dm	Eccentric-only	Eccentric-only resistance	10 weeks: 3 sessions/week	Week 1: 20–40% of	Borg Rating	22	Baseline	After 10 weeks	After 10 weeks, the	Missing or non-

	Qian et al (2024)/China [35]		resistance training group Age(51-66)	training including seated leg curl, leg press, lat pull-down, back extension, and arm flexion/extension exercises.		maximal strength (1 set, 15–30 min) Week 2: 30–50% of maximal strength (2 sets, 30–60 min) Weeks 3–10: 12-RM resistance training	of Perceived Exertion Scale (Borg RPE) and Visual Analogue Scale (VAS).		7.68 ± 1.43	7.05 ± 1.38	exercise group showed decreases in fasting glucose, insulin, insulin resistance, HbA1c, and triglycerides	extractable data<br>Inconsistent outcome reporting<br>Insufficient network connectivity
			Control Age (51-66)	Usual care				23	7.93 ± 1.70	7.77 ± 1.76		
23	Ranasinghe et al (2021)/Sri Lanka [41]	RCT/T2DM	RT Age (49.0 ±9.2)	7 major muscle group exercises (upper, lower, and core) using free weights, machines in a circuit format	12 weeks: 2 sessions/week, 60–75 min/session	50% RM, 3 × 8 repetitions with 5% increase in every 2 weeks	% RM	28	Baseline	After 12 weeks	HbA1c of RT vs. CN was 0.08% (95% CI, 0.8% to 0.7%, p ¼ 0.8) and AT vs. CN was 0.22% (95% CI, 0.95% to 0.5%).	Missing or non-extractable data<br>Inconsistent outcome reporting<br>Insufficient network connectivity
									7.6 ± 0.28	7.0 ± 0.24		
			Aerobic exercise training (AT) Age (52.0 ±9.8)	Circuit training with walking, stepping, and cycling	12 weeks: 2 sessions/week, 75 min/session.	60–75% of maximum heart rate (HRmax)	HRmax	28	8.1 ± 0.28	7.4 ± 0.24		
			Control Age (49.3 ±7.0)	Usual care	Standard clinic visits Telephone contact every 2 weeks to ensure continued participation			30	8.7 ± 0.28	8.2 ± 0.24		
24	Ranga et al (2021)/India [26]	RCT/ T2DM	Yoga Age (30-50)	A structured routine of postures (asanas) and relaxation	12 weeks: at least 5 times/week			50	Baseline	After 12 weeks	those who practiced yoga with medication had significantly lower fasting	Missing or non-extractable data
									8.08±0.90	7.88±0.85		

			Control Age (30-50)	No yoga exercises.	Only medication			50	8.54±0.90	8.42±0.91	glucose, HbA1c, and BMI than those who took medication only.	Inconsistent outcome reporting<br>Insufficient network connectivity
25	Sivapuram et al (2020)/India [27]	RCT/ T2DM							Baseline	After 12 weeks	HbA1c dropped significantly from 8.49% to 7.97% in Yoga group. After 3 months, their lipid profiles improved more than the control group ($p < 0.05$).	Missing or non-extractable data<br>Inconsistent outcome reporting<br>Insufficient network connectivity
			Yoga Age (58.86 ± 24.73)	Yoga postures, breathing exercises, meditation, relaxation, and health education.	12 weeks: Initial 9-day in-person camp: 2 hours/day of yoga. Followed by 1 hour/day home-based yoga practice. Weekly in-person follow-up sessions with a 2-hour review component.			50	8.49 ± 1.94	7.97 ± 2.20		
			Control Age (53.31 ± 7.71)	Received no yoga training or lifestyle counselling initially				31	8.60 ± 1.84	10.22 ± 1.66		
26	Sudarsono et al (2019)/Indonesia [43]	RCT/People with T2DM							Baseline	After 12 weeks	The combined HIIT and RT exercise program was not significantly improving glycemic control	Missing or non-extractable data<br>Inconsistent outcome reporting<br>Insufficient network connectivity
			HIIT combined with RT Age (51.69 ± 7.77)	Either a treadmill or an ergocycle.	12 weeks (frequency not reported)	HITT 1-2 weeks (60% - 70% Hrmax) 3-6 weeks (HIE: 90% HRmax; LIE: 70% HRmax) 7-12 weeks (HIE: 92% HRmax; LIE: 75%Hrmax) RT 1-2 weeks (adaptation phase) 3-6 weeks (external weight	HRmax for HIIT RPE for RT	18	7.96 ±1.11	7.53±0.73		

						resistance with RPE in week 5-6) 7-8 weeks (external weight resistance with RPE in week 7-8 9-12 weeks (external weight resistance with RPE in week 8-9)						
			Control Age (50.06 ± 7.19)	Continuous cardiorespiratory exercise performed once per week, with the same initial exercise volume as the experimental group.				15	8.02±1.67	7.70±1.32		
27	#*Viswanathan et al (2021)/ India {28]	RCT/ T2DM							Baseline	After 12 weeks	Yoga group lowers BMI, blood sugar, HbA1c, cholesterol, and inflammation markers.	
			Yoga Age (50.8 ± 8.3)	Loosening exercises, asanas, pranayama, and relaxation techniques, along with diabetes education	12 weeks: 5 days/week, 50 minutes/day			150	7.5 ±0.5	7.2 ±0.9		
			Non-Yoga Age (52.8 ± 7.0)	General physical activity	Do simple physical exercises for 50 min for 5 days a week.			150	7.5 ±0.6	7.6 ±1.1		

28	#*Way et al., (2020)/Australia [42]	RCT/T2DM	HIIT Age (56.9 ± 2.1)	Electronically braked upright cycle ergometer. Participants were asked to maintain their daily activity and diet pattern.	12 weeks: 3 sessions/week, 19 minutes/session	Each session with 4 minutes of high-intensity cycling at 90% VO_2peak	% of VO_2peak	12	Baseline<br>7.1 ±1.5	After 12 weeks<br>6.8 ±0.9	After the intervention of HIIT and MICT, significant improvements were found in VO_2peak ($p<.01$), HbA1c ($p = .03$), systolic blood pressure ($p< .01$), and waist size ($p = .03$).	
			MICT Age (54.8 ±2.4)	electronically braked upright cycle ergometer	12 weeks: 3 sessions/week, 55 minutes/session	Each session with 45 minutes of continuous cycling at 60% VO_2peak		12	7.3 ±1.5	7.0 ±1.1		
			Control Age (51.9 ±1.4)	Sham Exercise Placebo	Stretching and core exercises every 2 weeks; ≤30 min/session with 5-min light cycling (20 W) warm-up and cool-down.	Very light activity (20 watts)		11	7.6 ±0.5	8.0 ±0.5		
29	#Yamamoto (2021)/Japan [38]	Prospective RCT/T2DM	Resistance exercise (RE) Age (73.2 ± 2.6)	Using an elastic band and patients in RL receive leucine-rich suppl	48 weeks of daily exercise: one time/15 minutes	Bodyweight exercises combined with progressive elastic band resistance (TBB-1 to TBB levels), representing increasing resistance levels.	Daily exercise logs (adherence tracking).	18	Baseline<br>7.4 ±0.9	After 48 weeks<br>7.3±0.8	No changes in physical function, muscle mass, or cognitive function in any group. The leucine supplement brought no extra benefits for muscle strength or mass.	
			Control Age (73.3 ± 2.5)	Maintain daily activities	Receive no exercise and lifestyle modification			17	7.0 ±0.7	6.8 ±0.6		

Notes: # Articles included in preliminary meta-analysis, *Articles included in NMA., #* Articles included in both

Supplementary Table S3: Study effect and comparison-adjusted funnel plot

Study	Precision	Effect
Findikoglu et al. (2023) [36]	1.6	-0.32
Li et al. (2022) [31]	2.4	-0.42
Way et al. (2020) [32]	0.9	-0.71
Gupta et al. (2020) [25]	3.2	-0.21
Hirosaki et al. (2023) [37]	1.5	-0.32
Viswanathan et al. (2021) [28]	5.5	-0.4

Comparison-Adjusted Funnel Plot

Illustrative comparison-adjusted funnel plot for the network meta-analysis of HbA1c change.

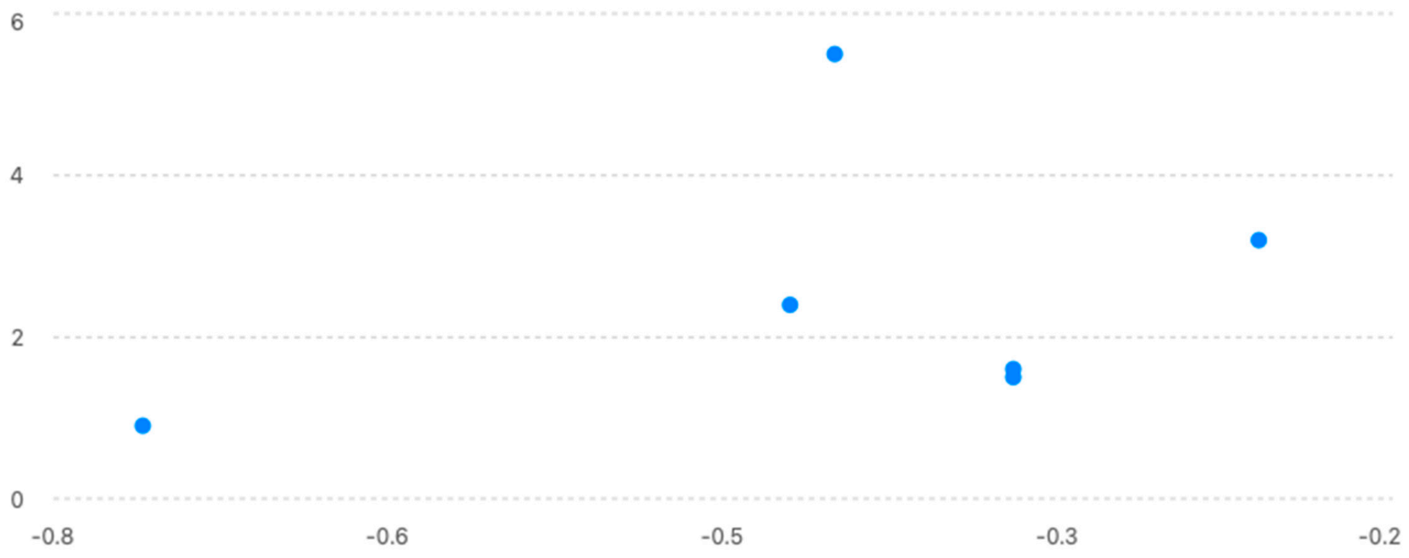

Smaller studies are located toward the bottom; larger studies appear near the top.

Legend: Comparison-adjusted funnel plot for the network meta-analysis of HbA1c change. Each point represents an individual study included in the network. Owing to the limited number of studies and absence of closed loops, the funnel plot was interpreted descriptively.

Supplementary Table S4: Arm-level data of studies included in NMA

Studies included in the NMA

Study	Treatment	N	Pre-intervention (mean±SD)	Post-intervention (mean±SD)	M Change	SD Change
Findikoglu et al. (2023) [36]	Active Control	20	6.99 ± 0.66	6.76 ± 0.66	-0.23	0.66
Findikoglu et al. (2023) [36]	HIIT	20	6.9 ± 0.68	6.59 ± 0.49	-0.31	0.6077006
Li et al. (2022) [31]	Active Control	12	7.06 ± 0.38	7.09 ± 0.33	0.03	0.3576311
Li et al. (2022) [31]	HIIT	13	7.18 ± 0.50	6.79 ± 0.41	-0.39	0.4616276

Way et al. (2020)	Active Control	11	7.6 ±0.5	8.0 ±0.5	0.4	0.5
Way et al. (2020)	HIIT	12	7.1 ±1.5	6.8 ±0.9	-0.312	1.3076697
Gupta et al. (2020) [25]	Active Control	41	8.39±0.65	8.38 ± 1.37	-0.01	1.1869709
Gupta et al. (2020) [25]	Yoga	40	8.53±0.71	8.31 ±1.32	-0.22	1.1442465
Hirosaki et al. (2023) [37]	Active Control	21	7.19 ± 0.7	7.26 ± 0.7	0.07	0.7
Hirosaki et al. (2023) [37]	Yoga	21	7.07±0.7	6.82 ± 0.6	-0.25	0.6557439
Viswanathan et al. (2021) [28]	Active Control	150	7.5 ±0.6	7.6 ±1.1	0.1	0.9539392
Viswanathan et al. (2021) [28]	Yoga	150	7.5 ±0.5	7.2 ±0.9	-0.3	0.781025

Notes: All HbA1c measurement units are in %
